# A primary care research agenda for multiple long-term conditions: a Delphi study

**DOI:** 10.3399/BJGP.2023.0163

**Published:** 2024-03-05

**Authors:** Jonathan Stokes, Peter Bower, Susan M Smith, Bruce Guthrie, Thomas Blakeman, Jose M Valderas, Chris Salisbury

**Affiliations:** MRC/CSO Social and Public Health Sciences Unit, School of Health and Wellbeing, University of Glasgow, Glasgow, UK; NIHR School for Primary Care Research, Centre for Primary Care and Health Services Research, University of Manchester, Manchester, UK.; NIHR School for Primary Care Research, Centre for Primary Care and Health Services Research, University of Manchester, Manchester, UK.; Department of Public Health and Primary Care, School of Medicine, Trinity College Dublin, Dublin, Ireland.; Advanced Care Research Centre, Usher Institute of Population Health Sciences, University of Edinburgh, Edinburgh, UK.; NIHR School for Primary Care Research, Centre for Primary Care and Health Services Research, University of Manchester, Manchester, UK.; Department of Family Medicine, National University Health System, Singapore; Centre for Research in Health System Performance, National University of Singapore, Singapore.; Centre for Academic Primary Care, Bristol Medical School, Population Health Sciences, University of Bristol, Bristol, UK.

**Keywords:** chronic disease, Delphi technique, developed countries, models of care, multimorbidity, primary health care

## Abstract

**Background:**

Multiple long-term conditions (MLTC), also known as multimorbidity, has been identified as a priority research topic globally. Research priorities from the perspectives of patients and research funders have been described. Although most care for MLTC is delivered in primary care, the priorities of academic primary care have not been identified.

**Aim:**

To identify and prioritise the academic primary care research agenda for MLTC.

**Design and setting:**

This was a three-phase study with primary care MLTC researchers from the UK and other high-income countries.

**Method:**

The study consisted of: an open-ended survey question, a face-to-face workshop to elaborate questions with researchers from the UK and Ireland, and a two-round Delphi consensus survey with international multimorbidity researchers.

**Results:**

Twenty-five primary care researchers responded to the initial open-ended survey and generated 84 potential research questions. In the subsequent workshop discussion (*n* = 18 participants), this list was reduced to 31 questions. The longlist of 31 research questions was included in round 1 of the Delphi; 27 of the 50 (54%) round 1 invitees and 24 of the 27 (89%) round 2 invitees took part in the Delphi. Ten questions reached final consensus. These questions focused broadly on addressing the complexity of the patient group with development of new models of care for multimorbidity, and methods and data development.

**Conclusion:**

These high-priority research questions offer funders and researchers a basis on which to build future grant calls and research plans. Addressing complexity in this research is needed to inform improvements in systems of care and for disease prevention.

## Introduction

Treating patients with multiple long-term conditions (MLTC), also known as multimorbidity, has been identified as a global research priority.^1^ Recent years have seen significant investment by public research funders to examine topics including clustering of multiple conditions,^2^^,^^3^ a priority-setting exercise on MLTC in later life from the patient’s perspective,^4^ and support from the third sector.^5^

Much of the current research on MLTC is at the ‘basic science’ stage, largely dealing with definitional issues, including which conditions should be counted,^6^^,^^7^ outlining the problems of having MLTC,^8^^–^10 and examining how conditions co-occur.^11^^,^^12^ Research in high-income countries is also at a different stage from that in low-income and middle-income countries,^13^ since the challenges currently faced are different. The hope is that better understanding of the current situation might point to (preventable) mechanisms and treatment groups to be prioritised.

There has been less focus on applied MLTC research: developing and testing new ways of working. The majority of primary care contacts are for patients with MLTC,^14^ and this research is of direct relevance to GPs and the wider workforce.^15^^,^^16^ As the only generalist discipline in many health systems, primary care is arguably the most important clinical discipline for this highly heterogeneous patient population. The academic primary care perspective, including academic clinicians and primary care researchers,^17^ is therefore vital for informing research strategy. This setting is also considered the bedrock of any integrated care across the wider system.^18^

Interventions trialled in this setting have achieved mixed results to date,^19^ and many uncertainties remain. The academic primary care community was therefore asked to provide their priorities for the future MLTC research agenda.

## Method

**Figure 1.**
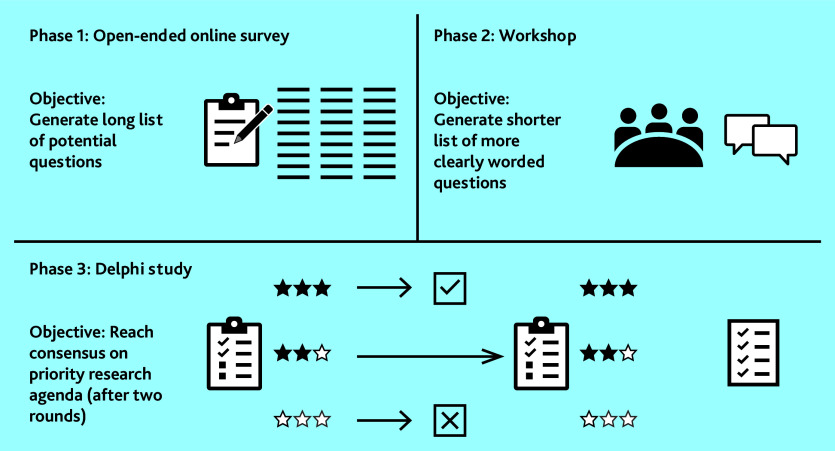
Outline of three-phase study design.

This was a three-phase study (open-ended survey question, face-to-face workshop, and a two-round Delphi study) with academic primary care MLTC researchers from the UK and other high-income countries, as outlined in Figure 1.

### Open-ended online survey

In early February 2022, each of the nine academic centres that make up England’s National Institute for Health and Care Research (NIHR) School for Primary Care Research (SPCR) were invited to nominate up to three representatives to take part. Those invited already had experience of researching MLTC, which improved the efficiency of the input.

**Table:** How this fits in

Research on multiple long-term conditions (MLTC) has been identified as a priority globally. To date, research and funding has focused on outlining and breaking down the problem of MLTC for the current system, with calls for applied solutions based predominantly in primary care. Primary medical care is responsible for most MLTC care, and this study draws on the expertise of academic primary care researchers to prioritise the applied research agenda for the next decade. These priorities offer funders and researchers a basis on which to build future grant calls and research plans.

Participants were asked to answer the question, ‘What are the big unanswered questions about MLTC (multimorbidity) in primary care that need to be addressed by the academic research community?’ Responses were not limited in number per participant, but were anonymous except for a final self-reported question on institutional affiliation. Participants were also given the option to forward the survey to colleagues who might be interested, and, in this way, experts from other parts of the UK and Ireland joined the process. The initial questions were then grouped into three overarching topic areas by the authors to structure workshop discussions.

### Workshop

The workshop was held face-to-face on 25 April 2022 in Manchester. Thirty-one researchers were invited, 20 (65%) of whom represented NIHR SPCR centres. It was held for 5.5 hours and was structured based on the topic areas identified from the open-ended survey. Each topic began with a short presentation from one of the project team. Then, the aim was to distil or expand the initial list of submitted questions through discussion, ordering, and rewording of printed copies. This shorter list of more clearly worded questions was the basis for the Delphi consensus study. The content of group discussions was captured with note-taking, as was the resulting prioritisation of the printed questions with any amendments made. The lead author summarised the discussion notes, and was checked for accuracy and representation of the discussions by three co-authors.

### Delphi study

Guidance on conducting and reporting Delphi studies was followed.^20^ The Delphi panel consisted of the original workshop invitees plus a purposively selected group of other ‘academic primary care researchers conducting MLTC research in high-income country settings’, which were the broad inclusion criteria applied. Researchers known to the authors were approached. PubMed was searched and screened for relevant abstracts and first authors using keywords for MLTC, primary care, and high-income countries, and reference lists from selected international systematic reviews were screened (see Supplementary Information).^21^^,^^22^ Finally, a call for volunteers was posted through the International Research Community on the Multimorbidity blog.^23^ In total, 50 participants were invited to the first round of the Delphi survey (*n* = 34, the 31 original workshop invitees plus three additional researchers known to authors; *n* = 5 responses to the blog; *n* = 11 from the literature).

Participants were asked to rate the questions in terms of their priority for the academic research community over the next 5–10 years. Rating was carried out using a Likert scale of 1–9, where 1 = not a research priority and 9 = highest priority for research. Participants were briefed that research areas to be prioritised might involve either qualitative or quantitative methods (or both) in practice. The research areas might also vary from more fundamental to more applied, and more specific research questions to more broadly defined areas of research interest. Regardless, they were asked to rank in terms of their overall priority. Round 1 also had an option to add any comments on the questions, and a final section to add additional question suggestions.

After round 1, participants were sent a summary report showing the status of each question (see Supplementary Information). Consensus was calculated given prespecified criteria. A score of 7–9 indicated endorsement, while a score of 1–3 indicated rejection. Questions that over 70% of participants endorsed, and less than 20% rejected, were deemed to have reached consensus. Questions that less than 40% endorsed, or over 20% rejected, were removed from round 2.^24^ All other questions, plus new suggestions or rewording after discussion among the co-authors, were re-evaluated in round 2. All surveys were conducted using the Qualtrics platform.

This study received a letter of ethical exemption from the University of Manchester, since it focused on asking professionals questions strictly within their professional competence, and not of a personal, sensitive, or confidential nature.

## Results

### Open-ended online survey

From the initial online survey, 84 questions were received from 25 responders at eight of the NIHR SPCR centres (84% of responders), plus four other institutions in the UK and Ireland. These questions were initially grouped into three topics for workshop discussions:

- models of care (the largest area, subdivided into
  - service delivery
  - organisation, back-office, and role of primary care);
- research methods/data; and
- outcomes (see Supplementary Information for full list).

### Workshop

Eighteen participants attended the workshop, 78% (*n* = 14) of whom were from NIHR SPCR centres. Discussion topics included the main differences between research on MLTC rather than single conditions, which participants felt were the complexity and variety of the patients encountered. This means there is less potential for standardisation of care, possibly requiring more flexibility and ownership for GPs and other clinicians to offer ‘holistic’ care. However, participants also recognised the likely trade-offs and paradigm shift of this type of care – for example, a possible move away from more guideline-based care, and potential knock-on effects for other system concerns such as waiting times (see Box 1 for further details of workshop discussions).

**Box 1.** Workshop discussions summary

**MLTC clusters**
In recognition of the tensions between system and patient demands, some kind of stratification or ‘clustering’ of patients was generally preferred by participants as a way of prioritising or differentiating care requirements. However, concerns about ‘actionable clusters’ were paramount, likely taking more of a biopsychosocial approach than currently prevalent in the MLTC clustering literature, where clusters were felt to be largely observational, with findings dependent on the specific statistical method employed. It was not always obvious how these clusters could then be employed to inform clinical practice and outcomes. For example, participants discussed potential for including social circumstances and needs rather than conditions alone. Even separating ‘risk factors’, from ‘symptoms’, from ‘complications’, from ‘individual conditions’ was not considered a straightforward task, either conceptually or using existing data (for example, for targeting specific clusters of patients or intervening at an appropriate point in a predicted trajectory). Improving understanding of clinically relevant clustering might require recording of socioeconomic and lifestyle factors that are currently lacking in many healthcare records, and there was concern about the time and resources available for collecting these data.
**Financial and non-financial incentives for managing MLTC**
Financial incentives were outlined as key for driving provider behaviour, including data collection and system changes supporting clinical practice, for example, extended consultations. Additionally, indicators and standards (for example, treatment targets and clinical guidelines) recorded at system level were perceived as having potentially helped to drive system change previously, particularly if led from a national regulatory body. There was therefore speculation as to whether it was possible to think about which set of indicators might be most useful to monitor and incentivise for MLTC patients.
**Primary care as part of management of MLTC across a wider system**
The theme of ‘complexity’ also carried through to discussions of interactions with the wider care system. Researching the role and amount of responsibility primary care should have in ensuring prevention, and how the primary care agenda should fit with other parts of the system, such as community assets, social prescribing, and the interface with secondary care, were also debated, together with the need to ensure continuity across different formal and informal sectors.
**Measuring appropriate outcomes for MLTC patients**
Similar complexity was also identified in terms of patient outcomes; for example, the potential for needs and goals to vary substantially between individual patients. The ability of conventional measures of benefit, such as health-related quality of life, to capture MLTC-relevant outcomes was questioned.
**Capturing complexity with appropriate study designs**
Optimal study designs were also considered, noting the need for a wider range of comparative study designs than randomised controlled trials that can adequately capture the complexity and variation. Multicomponent ‘system of care’ interventions with interacting elements, increasing use of observational quasi-experimental studies, ‘adaptive platform trials’, systems science approaches, and realist methods were all put forward as potential alternatives to deal with complexity.

*MLTC = multiple long-term conditions.*

Following discussion of the printed research questions, including rankings by small groups within the workshop, an executive group of the authors finalised the wording for 31 research questions for further consideration.

### Delphi study

Twenty-seven of the 50 invitees responded to the first Delphi survey (54% response rate). Participants included 10 (37%) males and 17 (63%) females from institutions based in the UK (*n* = 20), Ireland (*n* = 2), Canada (*n* = 1), Denmark (*n* = 1), France (*n* = 1), Singapore (*n* = 1), and the US (*n* = 1), a similar breakdown to those invited (see Supplementary Information). Sixteen (59%) represented NIHR SPCR centres. Seven of the 31 survey questions met the criteria for consensus after round 1. Four were removed from round 2. Twenty questions remained uncertain and were re-evaluated (five of these were reworded in response to written feedback). Three additional research questions were suggested by participants and introduced. Therefore, twenty-three research questions were evaluated in round 2.

Round 2 of the survey received responses from 24 of the 27 invitees (89% follow-up rate). Three further questions met the criteria for consensus, making a total of 10 (Table 1). An additional one question met the criteria for rejection. Nineteen questions remained uncertain (see Supplementary Information).

**Table 1.** Research questions that reached consensus at the end of the Delphi process

**Prioritised unanswered questions about MLTC (multimorbidity) in primary care for the academic research community over the next 5–10 years**	**Delphi consensus round**	**% endorsing (% rejecting)**	**Type of question**
1. What skill mix and training are needed for the primary care workforce to manage MLTC?	2	71% (8%)	Models of care
2. Which aspects of the current primary care model need to be adapted to improve management of MLTC?	1	74% (7%)	Models of care
3. How can we best develop care pathways for people with MLTC that are patient-centred and reduce burden of treatment?	1	81% (0%)	Models of care
4. How can primary care interventions contribute to prevention of disease onset or decline in function or quality of life?	2	79% (8%)	Models of care
5. How can primary care better interact with the wider health and social care system to improve MLTC care?	1	78% (4%)	Models of care
6. What are the barriers to implementation of potentially effective interventions?	1	89% (4%)	Models of care
7. How should MLTC be incorporated into primary care resource allocation models to better reflect the complexity of care and provide adequate resources?	1	78% (4%)	Methods and data
8. How can we improve the quality of data we gather from primary care for a better understanding of MLTC (e.g., disease severity, social factors, biological measures)?	1	74% (19%)	Methods and data
9. How can we make better use of administrative data and quasi-experimental observational studies to improve evaluation of MLTC interventions?	2	71% (8%)	Methods and data
10. What are the best ways of measuring wellbeing and quality of life for trials of MLTC interventions in primary care?	1	70% (7%)	Methods and data

*MLTC = multiple long-term conditions.*

The final list of prioritised questions can broadly be grouped into two categories: questions relating to researching new and adapted models of care, and questions relating to methods and data. Those dealing with models of care tended to have a higher proportion of responders endorsing prioritisation.

## Discussion

### Summary

A three-phase consensus study was conducted with academic primary care experts to identify priorities for MLTC research. Consensus was reached on 10 priority questions relating to development of new models of care for MLTC, and methods and data. These largely reflected the core theme of discussion in the workshop: the need to deal with the complexity of this patient group.

For models of care, priorities included both development and evaluation (of new models) and examination of implementation challenges. Priorities also included outcome measurement, such as a focus on experiential outcomes (patient-centred care and reducing treatment burden), and capturing upstream efforts for reducing negative outcomes (prevention of disease and functional decline or worsening quality of life). They reflected the complex challenge of re-orienting the wider health and care system. They also included, perhaps more immediately, challenges of adapting current models of care and skill mix, and identifying and addressing training needs of the primary care workforce.^25^

For methods and data, priorities reflected the need for these models to adequately resource (through funding formulas) the complexity of management. There was an identified need for new (or linked) data beyond what is commonly collected within primary care, to better capture determinants of outcomes and prevention opportunities. Finally, opportunities were identified to expand use of quasi-experiments and other evaluation methods to explore what is ultimately effective, which could require new measures of relevant outcomes.

### Strengths and limitations

The study used a three-stage approach to identify consensus research priorities of academic primary care. Limitations of this approach included the relatively small sample size and the modest response rate to the Delphi study. Nevertheless, the sample size is generally considered adequate for Delphi studies, where above 12 participants is likely to be subject to diminishing returns in terms of reliability.^26^ It was expected from the outset that the potential pool of researchers with expertise in the area would in any case be small overall. The follow-up rate in round 2 of the Delphi was high, so the sample was largely consistent between rounds. Another limitation is that responders were UK-centric, because of the funding and geographical location of the face-to-face workshop. Many of the UK participants were also affiliated with the NIHR SPCR, although this influence reduced over the phases (84% of open-ended survey responses, compared with 59% and 54% of round 1/2 Delphi responses). For all participants, researcher funding needs might have influenced responses. Nevertheless, the sample was expanded for the Delphi phase, and the final set of questions generated are likely to be relevant in many or most high-income countries. The final question set, however, does not necessarily represent all important issues in the topic area. There is an argument that creativity is what is needed, and new ideas will not necessarily reach consensus.

### Comparison with existing literature

These findings complement previous MLTC priority setting work, such as the James Lind Alliance (JLA) from the perspective of older patients,^4^ recommendations and research priorities from the Academy of Medical Sciences (AMS),^1^ and the UK’s NIHR strategic framework for MLTC.^27^ Areas of overlap and difference have been mapped in the Supplementary Information. Five of the 10 priority questions did not clearly overlap with any of the previous suggestions. Similarities shared across the findings and all these other exercises include a focus on prevention of disease onset (or further decline, priority number 4), and the need for a coordinated response to (integrated) care across the whole health and care system (priority number 5). The AMS priorities further overlapped in recognising a need for strategies to improve MLTC management (although not specifically in primary care, priority number 2). The NIHR priorities also overlapped with the need for patient-centred care pathways (priority number 3) and to augment outcome measures to those most relevant to patients (similar to priority number 10).

In contrast with the priority findings identified here, the JLA’s priorities tended to emphasise specific outcome priorities for older patients (for example, social isolation, psychological wellbeing, independent living, and risk of falls), and specific intervention areas (for example, supporting carers, exercise therapy, and Comprehensive Geriatric Assessment). Arguably, several of these also emphasised a change to organisation of care to deliver the specific outcome or intervention.^4^ The AMS, on the other hand, had three priorities related specifically to clusters of conditions that did not come up in this exercise: trends and patterns (also reflected in the NIHR priorities),^27^ identifying clusters causing the greatest burden, and their determinants.^1^ Academic primary care researchers, particularly in this study’s workshop, raised potential limitations with the clinical relevance and application of cluster studies. For example, it is questionable how clusters might inform any attempt to reduce secondary care costs, where a large number of co-occurring conditions each contribute to a very small fraction for MLTC patients.^28^

Unlike these previous exercises, academic primary care researchers, understandably, also prioritised several methods and data questions. These addressed complexity and problems in adequately developing, evaluating, and implementing improved models of care, and being able to judge whether ‘effectiveness’ has been achieved (priorities 6, 8, and 9, which did not feature in previous exercises). Additionally, and perhaps reflecting the current pressures on healthcare systems, the specific emphasis on workforce skill mix and training (priority number 1), and resource allocation decisions (priority number 7) to support MLTC care have not featured previously.^25^ Ensuring these practical aspects receive attention is likely to be important for impact of research funding, policymakers, practitioners, and, ultimately, patient outcomes.

As outlined above, the priorities generated also differ quite substantially from the bulk of funded and published MLTC research to date. This perhaps also reflects the stage of the current MLTC evidence base, where the main gap is now application to build on the basic research already conducted.

### Implications for research and practice

These priorities can offer funders and researchers a basis on which to build future grant calls and research plans. However, as raised in the workshop, the nature of the competitive scientific funding process might create challenges in obtaining support for novel, potentially groundbreaking, research in this area, as there might be a real or perceived higher level of risk.^29^ For example, there is the tension between allowing clinicians to recruit potentially eligible patients for interventions using the clinical expertise and judgement they apply in routine care, and concerns about selection bias or risks of using novel methods and data collection. Aligning research funding to the exploration of complexity might be the next frontier, and is likely to require context-specific solutions. Seeking to ignore complexity with traditional research methods that minimise variation in participants, intervention, implementation, and outcomes is no longer an option for improving systems of care or for disease prevention.^30^
